# Toward a Consensus in the Repertoire of Hemocytes Identified in *Drosophila*

**DOI:** 10.3389/fcell.2021.643712

**Published:** 2021-03-04

**Authors:** Pierre B. Cattenoz, Sara Monticelli, Alexia Pavlidaki, Angela Giangrande

**Affiliations:** ^1^Institut de Génétique et de Biologie Moléculaire et Cellulaire, Illkirch, France; ^2^Centre National de la Recherche Scientifique, UMR 7104, Illkirch, France; ^3^Institut National de la Santé et de la Recherche Médicale, U1258, Illkirch, France; ^4^Université de Strasbourg, Illkirch, France

**Keywords:** macrophage, single cell RNA seq, drosophila, lamellocyte, innate immunity

## Abstract

The catalog of the *Drosophila* immune cells was until recently limited to three major cell types, based on morphology, function and few molecular markers. Three recent single cell studies highlight the presence of several subgroups, revealing a large diversity in the molecular signature of the larval immune cells. Since these studies rely on somewhat different experimental and analytical approaches, we here compare the datasets and identify eight common, robust subgroups associated to distinct functions such as proliferation, immune response, phagocytosis or secretion. Similar comparative analyses with datasets from different stages and tissues disclose the presence of larval immune cells resembling embryonic hemocyte progenitors and the expression of specific properties in larval immune cells associated with peripheral tissues.

## Introduction

Immune cells are able to move and connect distant tissues and organs. This feature likely accounts for their pleiotropic role as sensors and regulators of the internal state in homeostatic, challenged and pathological conditions. While pleiotropy seems to arise from immune cell heterogeneity, the cause and nature of cell diversity is still poorly understood. How much does it depend on intrinsic differences dictated by cell autonomous cues vs. environmental conditions met by these cells during their life? To address the longstanding question on the impact of nature vs. nurture, of cell identity vs. cell state, we first need to characterize the different subtypes in depth.

Beside a lower complexity of the immune cell lineages, *Drosophila* shares with vertebrates several factors controlling the differentiation of the myeloid lineage [e.g., GATA and Runx proteins (Wood and Jacinto, 2007)], immune cell migration [e.g., integrins and Rho GTPases (Paladi and Tepass, 2004; Siekhaus et al., 2010; Comber et al., 2013)], phagocytosis [e.g., the CED-1 family member Draper and the CD36-related receptor Croquemort (Franc et al., 1996; Manaka et al., 2004)] and immune response [i.e., JAK/STAT, IMD, and Toll pathways (Buchon et al., 2014)]. Hence, it represents a simple yet evolutionary conserved model to address the origin of the immune cell diversity.

The immune cells of *Drosophila*, the hemocytes, have been classically subdivided in three types: the plasmatocytes, the crystal cells and the lamellocytes, which are thought to derive from the same lineage, similar to the myeloid cells in mammals (Banerjee et al., 2019). The plasmatocytes are macrophage-like cells that phagocytose pathogens as well as cell debris and constitute ∼95% of the hemocytes. The remaining hemocytes are the crystal cells, platelet-like cells in charge of melanization, a process that is necessary for wound closure and immune response to pathogens. The third type of immune cells, the lamellocytes, appears only after immune/inflammatory challenge from progenitors or by plasmatocyte transdifferentiation (Banerjee et al., 2019).

Until recently, the hemocytes were identified using distinctive morphological features and a handful of molecular markers. The plasmatocytes are small cells of ∼10 μm of diameter. They can be round or present cytoplasmic projections [i.e., podocytes (Rizki and Rizki, 1980)]. They express several markers such as the transmembrane receptors Nimrod C1 (NimC1), Eater, Hemese (He) and Croquemort (Crq), the fascin Singed (Sn) and the secreted proteins Hemolectin (Hml), Peroxidasin (Pxn), and Collagen type IV alpha 1 (Col4a1) (Nelson et al., 1994; Franc et al., 1996; Goto et al., 2001; Kurucz et al., 2003; Kocks et al., 2005; Zanet et al., 2009). The crystal cells have the same size than the plasmatocytes and are characterized by the presence of crystals. They express the transcription factors Lozenge (Lz) and Pebbled (Peb) as well as Prophenoloxidases 1 and 2 (PPO1 and PPO2) (Rizki and Rizki, 1959; Binggeli et al., 2014). PPO2 is a major constituent of the crystals, which are released upon wounding to initiate the melanization reaction (Binggeli et al., 2014). The lamellocytes are large melanized cells (>60 μm of diameter) with heterogeneous shapes. They are strongly labeled with the actin filament probe called phalloidin and express the Prophenoloxidase 3 (PPO3), the kinase Misshapen (Msn), the integrins Myospheroid (Mys, Integrin beta or L4), and Integrin alphaPS4 subunit (ItgaPS4 or L5), the actin binding protein Cheerio (Cher, L5), and the glycosylphosphatidylinositol (GPI)-anchored protein Atilla (L1) (Braun et al., 1997; Irving et al., 2005; Rus et al., 2006; Honti et al., 2009).

The development of single cell RNA sequencing (scRNAseq) techniques has made it possible to significantly enlarge the panel of the *Drosophila* immune cells based on their transcriptional profile. ScRNAseq consists of sequencing the transcriptome of single cells in a high throughput fashion. The cells are then grouped according to their expression profiles (reviewed in Potter, 2018; See et al., 2018). Three scRNAseq studies recently revealed the diversity of the hemocytes present in the *Drosophila* larva (Cattenoz et al., 2020; Fu et al., 2020; Tattikota et al., 2020). We perform here a comparative study to refine immune cell diversity, origin and localization within the organism. Our comparison defines subgroups robustly found in the three datasets from steady state larval hemocytes, despite the different experimental and analytical approaches. The common subgroups reflect the differentiation state, intermediary vs. mature hemocytes, as well as their main functions (phagocytosis, immune response/antimicrobial peptide (AMP) production, secretion and proliferation). Finally, we analyze the hemocytes present in available single cell datasets from larval eye discs and brains as well as from stage 6 embryos in order to link specific subgroups to distinct environments/developmental trajectories.

## Materials and Methods

### Comparison of scRNAseq Data on Wandering 3rd Instar Larval Hemocytes

The list of markers for each subgroup were retrieved from the publications [Dataset_EV2 in Cattenoz et al. (2020) and Supplementary File 2 in Tattikota et al. (2020)]. The two lists were generated with the same tool [function “FindMarkers” in Seurat R toolkit (Butler et al., 2018; Stuart et al., 2019)] and provide comparable parameters including the enrichment levels for all the subgroup markers. The two tables were compiled in R and plotted using the package ggplot2 (Villanueva and Chen, 2019). The markers described in Fu et al. (2020) dataset were retrieved from the figure of the manuscript. Of note, the PM12 cells in Tattikota et al. (2020) appear exclusively in wounding condition, an experimental set up that was not assessed by the two other studies. To keep the comparison as homogenous as possible, the markers of the subgroup PM12 were excluded from our analysis.

The dot plots were generated using the function “DotPlot” in the Seurat R toolkit (Butler et al., 2018; Stuart et al., 2019) with the non-infested data from Cattenoz et al. deposited in the ArrayExpress database at EMBL-EBI^1^ under the accession number E-MTAB-8698. The list of markers for the CAH7 PM, the Lsp PM, the Ppn PM, the thanacytes, and the primocytes were retrieved from the figures in Fu et al. (2020). The top PSC markers were retrieved from the lymph gland scRNAseq data from Cho et al. (2020; Supplementary Table S2): the markers presenting the highest enrichment in the PSC were selected.

### Comparison of the WL Hemocyte scRNAseq Data With Stage 6 Embryo, Larval Eye Disc and Larval Brain scRNAseq Data

The normalized expression matrix of the stage 6 embryos (Karaiskos et al., 2017) was downloaded from https://shiny.mdc-berlin.de/DVEX/ and analyzed with the standard workflow from Seurat toolkit^2^ (Butler et al., 2018; Stuart et al., 2019). Briefly, first the data were normalized (function NormalizeData), the variable genes were identified (function FindVariableFeatures, selection method “vst,” number of feature = 2,000), the data were scaled (function ScaleData, features = all.genes), linear dimensional reduction was carried out on the variable genes (function RunPCA), the dimensionality of the dataset was determined and set to 15 (function ElbowPlot), the cells were then clustered (functions FindNeighbors, dims = 1:15 and FindClusters, resolution = 1.2), at last non-linear dimensional reductions were carried out (functions RunUMAP and RunTSNE). This pipeline generated a single subgroup enriched for all the markers of the hemocyte subgroup described in Karaiskos et al. (2017): i.e., Gcm, Ham, Ttk, CrebA, Shep, RhoL, Fok, Knrl, Kni, Zfh1, CG33099, Srp, Btd, and NetB. This subgroup was used for the downstream analyses.

The normalized expression matrix of the wild type larval eye disc (Ariss et al., 2018) was downloaded from https://www.ebi.ac.uk/gxa/sc/experiments/E-MTAB-7195/downloads and analyzed following the same pipeline than stage 6 embryos described above with modification: the dimensionality was set to 17 and the cells were clustered with a resolution of 0.5. The hemocyte subgroup was unambiguously identified using the hemocyte markers Srp, Hml, Pxn, NimC1, Crq, and Sn and used for the downstream analyses.

The expression matrices of the normal 1st instar larval brains (Brunet Avalos et al., 2019) were downloaded from https://www.ncbi.nlm.nih.gov/geo/query/acc.cgi?acc=GSE134722. The expression matrices GSM3964166, GSM3964167, GSM3964168, and GSM4132287 were merged and integrated following Seurat standard pipeline (Butler et al., 2018; Stuart et al., 2019): each matrix was normalized (function NormalizeData), the variable features and the common anchors were identified (function FindVariableFeatures, method “vst,” function FindIntegrationAnchors, dimension 1:50) and the matrices were integrated (function IntegrateData, dimension: 1:50). The integrated matrix was analyzed following the same pipeline as described above for the stage 6 embryos with the following parameters: the dimensionality was set to 50 and the cells were clustered with a resolution of 0.4. The hemocyte subgroup was unambiguously identified using the hemocyte markers Srp, Hml, Pxn, NimC1, and He and used for the downstream analyses.

The expression matrices of the brains from 2nd instar larvae [24 h after larval hatching (h ALH)], feeding 3rd instar larvae (48 h ALH) and wandering 3rd instar larvae (96 h ALH) (Cocanougher et al., 2019) were downloaded from https://www.ncbi.nlm.nih.gov/geo/query/acc.cgi?acc=GSE135810. The following matrices were used: GSM4030602, GSM4030604, and GSM4030607 for the 2nd instar larvae, GSM4030600 and GSM4030606 for the feeding 3rd instar larvae and GSM4030623, GSM4030624, GSM4030625, and GSM4030626 for the wandering 3rd instar larvae. The matrices were integrated for each stage and analyzed as described for the 1st instar larval brain described above with the following parameters: the dimensionality was set to 30 and the cells were clustered with a resolution of 0.4 for the 2nd instar larvae and 2.4 for the feeding and wandering 3rd instar larvae. The hemocyte subgroups were unambiguously identified using the hemocyte markers Srp, Hml, Pxn, NimC1, He, and Nplp2 (described by the authors) and used for the downstream analyses.

Pearson correlation were computed as follow. Pseudo-transcriptomes were generated for the hemocyte subgroups from the stage 6 embryos data (Karaiskos et al., 2017), from the eye disc data (Ariss et al., 2018), for the brain data (Brunet Avalos et al., 2019) as well as for each subgroup of the non-infested dataset from Cattenoz et al. (2020) using the function “AverageExpression” from the Seurat R toolkit (Butler et al., 2018; Stuart et al., 2019). The correlation between the pseudo-transcriptomes were then measured using the Pearson correlation coefficient. The pseudo-transcriptomes of the hemocyte subgroups from Cattenoz et al. were compared to the pseudo-transcriptome of stage 6 embryos’ hemocytes in Supplementary Figure S3G, to the pseudo-transcriptome of the eye disc associated hemocytes in Supplementary Figure S3C and to the pseudo-transcriptome of the brain associated hemocytes in Supplementary Figure S3E.

The dot plot (Figure 4D) was generated using the function “DotPlot” from the Seurat R toolkit with the non-infested data from Cattenoz et al. (2020) and the expression matrices from stage 6 embryos and larval eye discs (Karaiskos et al., 2017; Ariss et al., 2018). The dot plot was compiled in Adobe Illustrator CS6.

### Regulon Analysis

To identify the regulons enriched in the lamellocytes, we ran Single-Cell regulatory Network Inference and Clustering (SCENIC) (Aibar et al., 2017) through its Python implementation pySCENIC, version 0.9.19^3^. The source code was downloaded from the GitHub repository https://github.com/aertslab/pySCENIC.git. The Supplementary Files necessary to run SCENIC were obtained from https://resources-mirror.aertslab.org/cistarget/. The analysis was carried out on the wasp infested expression matrix from Cattenoz et al. deposited in the ArrayExpress database at EMBL-EBI^4^ under the accession number E-MTAB-8698.

The motifs version 8,^5^ the regulatory elements within 5 kb upstream the TSS and the transcript introns^6^ were used for the analysis. The most significant regulons showing differential activity among clusters were determined with Mann-Whitney *U*-test (Mann and Whitney, 1947), between the AUC scores given by SCENIC in a specific cluster versus all the rest of the clusters. The regulons displaying a *z*-score above 2 or below −2 for the lamellocytes subgroups were selected to build the heatmap shown in Figure 3A. The heatmap was generated with the R package “pheatmap” (Kolde, 2019).

The scatter plot (Figure 3B) was generated with the pseudo-transcriptomes of LM1 and LM2 subgroups from the wasp infested dataset in Cattenoz et al. (2020). The pseudo-transcriptomes were estimated with the function “AverageExpression” from the Seurat R toolkit (Butler et al., 2018; Stuart et al., 2019).

### Fly Strains and Genetics

All flies were raised on standard media at 25°C. The following strains were used: *Oregon-R, srp(hemo)-3xmcherry* [*srp(hemo)* > *RFP*, gift from D. Siekhaus (Gyoergy et al., 2018)], *BAC-gcm-Flag* (Laneve et al., 2013).

### Immunolabelling and Image Acquisition

For hemocyte labeling, 10 wandering 3rd instar larvae were bled in Schneider medium complemented with 10% Fetal Calf Serum (FCS), 0.5% penicillin, 0.5% streptomycin (PS), and few crystals of N-phenylthiourea ≥98% (PTU). The cells were cytospinned on a glass slide at 700 rpm for 3 min at room temperature (RT), then the samples were fixed for 10 min in 4% paraformaldehyde/PBS at RT and rinsed with PTX (PBS 1x, 0.5% triton X-100).

For the embryos, overnight collections were washed on a 100 μm mesh and dechorionated in bleach for 5 min. The fixation was carried out for 25 min at RT under agitation in a solution of 4% paraformaldehyde in PBS 1x/heptane (1/1 vol.). The vitelline membrane of the embryos was then removed by replacing the PFA solution by methanol and strong agitation for 30 s. The methanol/heptane solution was removed and the embryos were washed with PTX for 15 min at RT.

For the lymph gland and filet preparation, wandering 3rd instar larvae were dissected in cold PBS 1x, then transferred in 4% paraformaldehyde in PBS 1x for at least 30 min at RT and rinsed in PTX for 15 min.

Following the PFA fixation and PTX wash, the samples were incubated with blocking reagent (Roche) for 1 h at RT, incubated overnight at 4°C with primary antibodies diluted in blocking reagent, washed three times for 10 min with PTX, incubated for 1 h with secondary antibodies, washed twice for 10 min with PTX, incubated for 30 min with DAPI and phalloidin TRITC (1:1,000, Sigma #P1951), and then mounted in Aqua-Poly/Mount (Polysciences, Inc.). The following primary antibodies were used: rabbit anti-Srp [1:500, (Bazzi et al., 2018)], rabbit anti-Flag (1:100, Sigma S3165), chicken anti-GFP (1:500, abcam ab13970), rat anti-RFP (1:500, Chromotek 5F8-100), rabbit anti-Pxn [1:5,000; gift from J. Shim, (Yoon et al., 2017)], mouse anti-Hemese [1:50 gifts from I. Ando, (Kurucz et al., 2003)].

The following secondary antibodies were used at 1:500: FITC donkey anti-chicken IgG (Jackson ImmunoResearch Labs Cat# 703-095-155), FITC goat anti-mouse IgG (Jackson ImmunoResearch Labs Cat# 115-095-166), Cy3 donkey anti-mouse IgG (Jackson ImmunoResearch Labs Cat# 715-165-151), Cy3 goat anti-rat IgG (Jackson ImmunoResearch Labs Cat# 112-165-167), Cy3 donkey anti-rabbit IgG (Jackson ImmunoResearch Labs Cat# 711-165-152), Cy5 goat anti-mouse IgG (Jackson ImmunoResearch Labs Cat# 115-175-003) and Cy5 goat anti-rat IgG (Jackson ImmunoResearch Labs Cat# 112-175-167), Cy5 goat anti-rabbit IgG (Jackson ImmunoResearch Labs Cat# 111-175-144), Alexa Fluor 647 goat anti-mouse IgG (Jackson ImmunoResearch Labs Cat# 115-605-166).

The slides were analyzed by confocal microscopy (Leica Spinning Disk and Leica SP8) with 20x, 40x, and 63x objectives, using hybrid detectors in photon counting mode. DAPI was excited at 350 nm, the emission filters 410–510 were used to collect the signal; FITC was excited at 488 nm, the emission filters 498–551 were used to collect the signal; Cy3 was excited at 568 nm, emission filters 648–701 were used to collect the signal, and Cy5 was excited at 633 nm; emission signal was collected at 729–800 nm. The images were analyzed with Fiji (Schindelin et al., 2012).

## Results

### Characterization of the *Drosophila* Larval Hemocytes by scRNAseq

**FIGURE 1:**
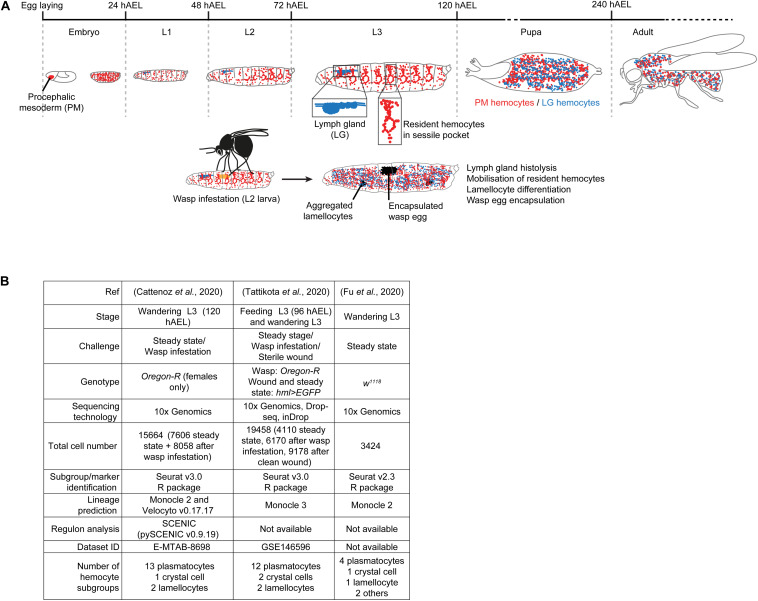
Experimental procedures used to generate the scRNAseq datasets on larval hemocytes. **(A)** Overview of the two hematopoietic waves during *Drosophila* development. The first wave starts in the procephalic mesoderm (PM) of the early embryo. By the end of embryogenesis, the PM hemocytes spread across the whole embryo and constitute the whole population of hemocytes present in the hemolymph of the larva. By the 2nd larval instar, the majority of the PM hemocytes become resident between the muscle and the cuticle to form the sessile pockets. After pupariation, the hemocytes are mobilized in the hemolymph. The second wave occurs in the lymph gland (LG) during the larval stages, the LG hemocytes being released in circulation after pupariation. When the wasp infests the larva (2nd instar), it induces a strong immune response, which includes precocious lymph gland histolysis and the release of the LG hemocytes in 3rd instar larvae. The response also includes the mobilization of the resident hemocytes and the differentiation of lamellocytes to encapsulate the wasp egg. The lamellocytes spontaneously aggregate and form melanized masses called melanotic tumors. The developmental stages and their duration at 25°C are indicated in gray in hours After Egg Laying (hAEL). **(B)** Experimental parameters of the three single cell sequencing analyses of PM hemocytes.

The first hematopoietic wave occurs at early embryonic stages 6–9, in the procephalic mesoderm (PM). The progenitors undergo several rounds of division, differentiate into plasmatocytes and crystal cells and migrate along stereotyped routes to spread throughout the organism (PM hemocytes) (Tepass et al., 1994; Gold and Bruckner, 2014). The second hematopoietic wave occurs in the lymph gland (LG) of the larva to generate cells that are only released in the hemolymph after puparium formation, upon lymph gland histolysis (LG hemocytes) (Jung et al., 2005; reviewed by Banerjee et al., 2019). The larval infestation from parasitoid wasps such as *Leptopilina boulardi* as well as wounding triggers precocious lymph gland histolysis. These challenges also lead to the differentiation of lamellocytes that encapsulate the wasp eggs or participate to the wound closure (Figure 1A) and the same cell type is found upon the activation of pro-inflammatory pathways (Lemaitre et al., 1995; Luo et al., 1995; Markus et al., 2005; Fleury et al., 2009; Kim-Jo et al., 2019).

The larval PM hemocytes were recently analyzed in three scRNAseq studies (Cattenoz et al., 2020; Fu et al., 2020; Tattikota et al., 2020), following different experimental parameters (Figure 1B). Tattikota et al. analyzed hemocytes from feeding 3rd instar larvae and wandering 3rd instar larvae (WL) in steady state conditions or after inducing an immune reaction (clean wounding, or wasp infestation). Three sequencing technologies were used and the animals were of two genotypes. The merge of these datasets identified twelve subgroups of plasmatocytes, two of crystal cells and two of lamellocytes (Tattikota et al., 2020). Fu et al. analyzed hemocytes from WL of one genotype in steady state conditions using 10x Genomics technology. They identified four subgroups of plasmatocytes, one of crystal cells, one of lamellocytes and two minor cell populations called primocytes and thanacytes (Fu et al., 2020). Cattenoz et al. analyzed hemocytes from female WL of one genotype, in steady state conditions and after wasp infestations, using 10x Genomics technology. They identified thirteen subgroups of plasmatocytes, one of crystal cells and two of lamellocytes, the latter ones being specifically found in the dataset from the challenged condition (Cattenoz et al., 2020). In the three datasets, the definition of the subgroups was followed by lineage prediction analyses to identify distinct developmental trajectories amongst the hemocyte subgroups. The trajectories are defined by organizing the cells along a pseudo-time axis based on the progression of the expression of the variable genes (Kester and van Oudenaarden, 2018).

**FIGURE 2:**
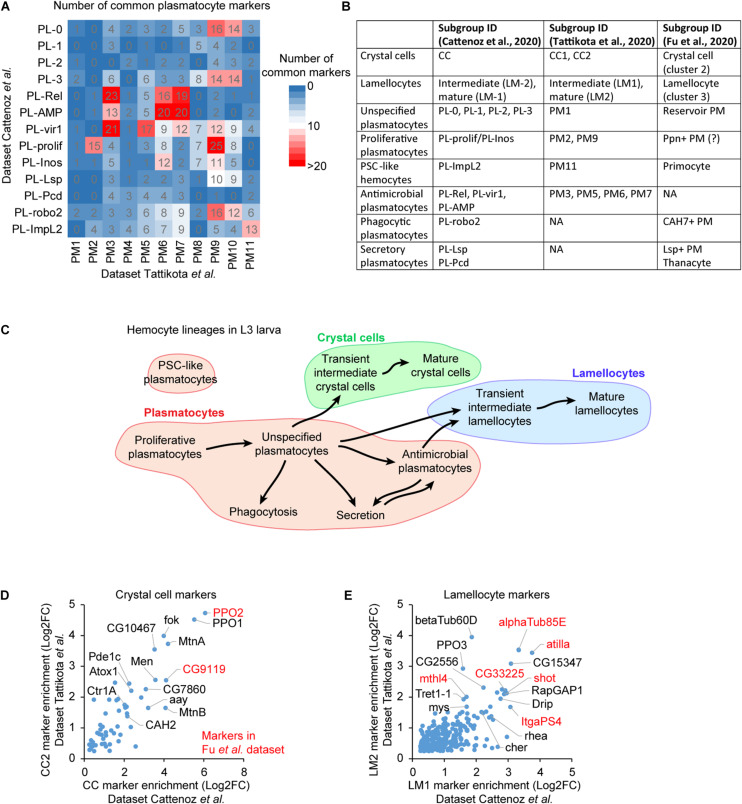
Defining a consensual repertoire of the hemocyte subgroups. **(A)** Number of common markers between the plasmatocyte subgroups identified in Cattenoz et al. (2020) (rows) and in Tattikota et al. (2020) (column). The number of markers is highlighted by the color gradient (from 0 in blue to >20 in red). **(B)** Concordance amongst the hemocyte subgroups across the three datasets. **(C)** Outline of the lineage predictions among the hemocyte subgroups, based on the prediction analyses in Cattenoz et al. (2020) and Tattikota et al. (2020) and on the present comparative analysis. The proliferative plasmatocytes are at the origin of most hemocytes, they give rise to a large pool of unspecified plasmatocytes that further differentiate to acquire specific properties such as phagocytosis, secretion or antimicrobial peptide production. Subsets of unspecified plasmatocytes and antimicrobial plasmatocytes transdifferentiate to produce crystal cells or lamellocytes. **(D)** Scatter plot representing the enrichment levels of the crystal cell markers common to the subgroups CC2 (y-axis) from Tattikota et al. (2020) and CC (x-axis) from Cattenoz et al. (2020). The Log2 fold change (Log2FC) of the enrichment is represented. The markers with the strongest enrichment are indicated on the graph, those also identified in Fu et al. (2020) are highlighted in red. **(E)** Scatter plot representing the enrichment of the lamellocyte markers in the indicated subgroups. The representation is as in panel **(D).**

The systematic comparison of the lists of markers from the three studies allows the identification of common subgroups (Figure 2A). The markers are defined by their levels of enrichment in a specific subgroup compared to the whole hemocyte population [Log2(enrichment) >0.25, adjusted *p* < 0.01 in Cattenoz et al. (2020) and Tattikota et al. (2020)]. The correlation between the subgroups of each dataset is inferred from the cross-comparison of all plasmatocyte markers found in the three studies (Figure 2A). The number of markers as well as their level of enrichments are taken into consideration (Supplementary Figures S1A,B, S2A,C–E). An additional level of comparison among the datasets is the developmental trajectory. If our comparison based on markers is accurate, the developmental trajectories between the distinct subgroups should be preserved across the three studies, and we find that this is the case. Thus, the subgroups presenting the highest number of common markers, the highest levels of markers’ enrichment and similar developmental trajectories likely define equivalent subgroup (Figures 2B,C).

**FIGURE 3:**
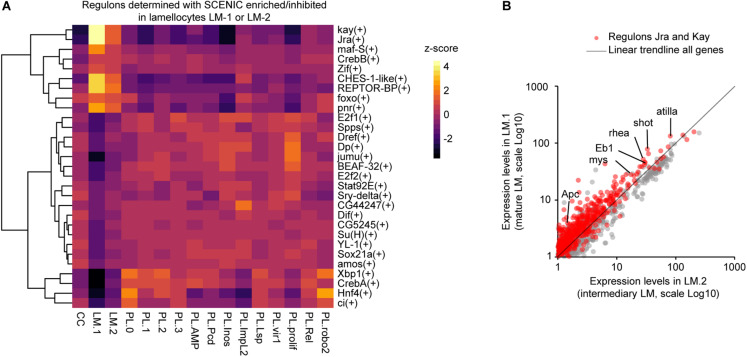
Characterization of the regulatory network controlling lamellocyte differentiation. **(A)** Heatmap representing the z-score for the regulons enriched or underrepresented in the lamellocyte subgroups (*z*-score >2 or < -2) determined with SCENIC. The dendrogram on the left side of the panel indicates the correlation between the regulons across the dataset. The *z*-score is indicated with a gradient from black (*z*-score < 0, underrepresented regulons) to red and then yellow (*z*-score > 0, overrepresented regulons). **(B)** Scatter plot comparing the pseudo-transcriptomes of mature lamellocytes (y-axis) and transient intermediary lamellocytes (x-axis). The pseudo-transcriptomes were generated from the dataset obtained upon wasp infestation (Cattenoz et al., 2020). The genes belonging to the regulons Jra and Kay are indicated in red, the trendline of all genes expressions is in black, the axes are in Log10 scale.

**FIGURE 4:**
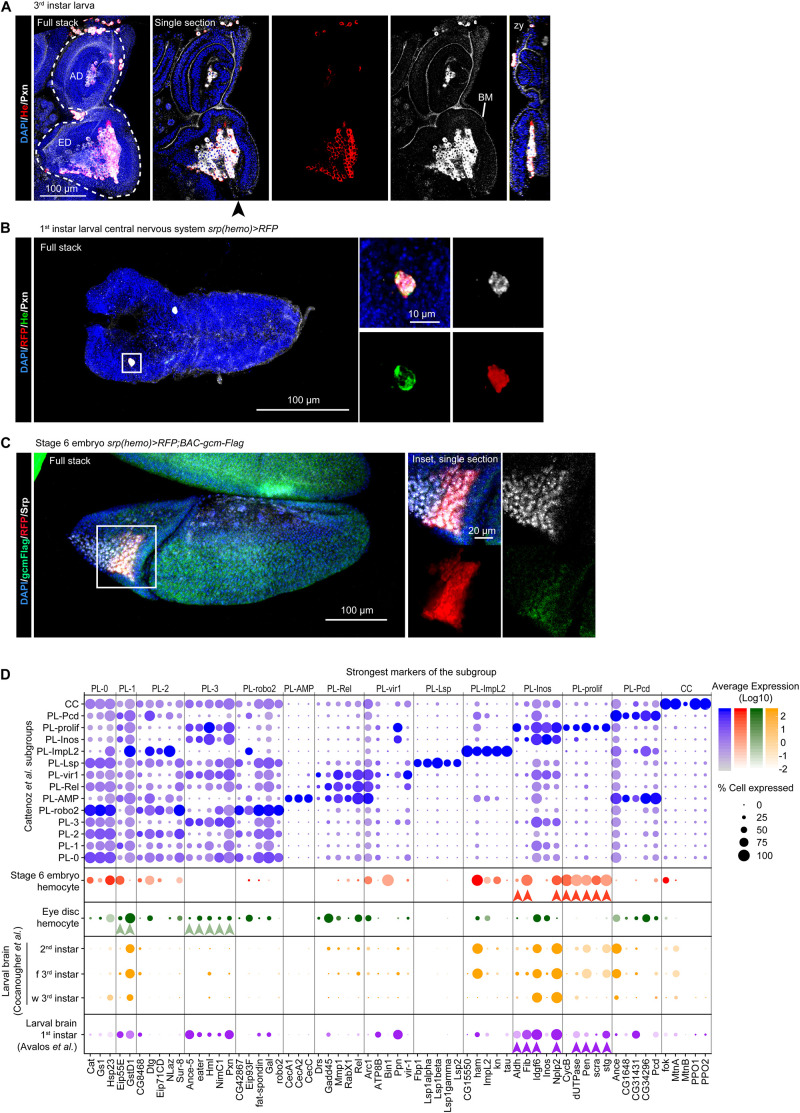
The molecular signatures of the hemocytes from larval eye discs, larval brains and stage 6 embryos. **(A)** Eye disc from 3rd instar larva. The immunolabelling assay used anti-He (in red), anti-Pxn (in white) and the nuclei were labeled with DAPI (blue). The left panel shows the full stack, the eye disc (ED) and the antenna disc (AD) are indicated with a white dashed line, the next panels show a single section with all the channels, He labeling only and Pxn labeling only, respectively. Pxn labeling is present at the basement membrane (BM). The rightmost panel shows an orthogonal projection along the z-y axes from the position indicated by the black arrowhead. The scale bar represents 100 μm. **(B)** Central Nervous System from 1st instar larva carrying the *srp(hemo) > RFP* transgene, which allows for hemocyte detection (Gyoergy et al., 2018). The immunolabelling assay used three hemocyte markers [anti-RFP (in red), anti-He (in green), anti-Pxn (in gray)], and the nuclei were labeled with DAPI (blue). The left panel shows the full stack, the right panels (partial stacks) show the inset at high magnification (merged and individual channels). The scale bars represent 100 μm in the full stack and 10 μm in the inset. The hemocyte lays on the surface of the nervous system. **(C)** Stage 6 embryo *srp(hemo) > RFP; BAC-gcm-Flag*. The BAC was used to reveal the Gcm protein (Laneve et al., 2013). The immunolabelling assay used anti-RFP (in red), anti-Flag (in green), anti-Srp (in gray), and the nuclei were labeled with DAPI (blue). The left panel shows the full stack, the right panels show a single section of the inset at high magnification (merged and individual channels). The scale bars represent 100 μm in the stack and 20 μm in the inset. **(D)** Dot plot representing the expression levels and percentage of cells expressing the top markers of each subgroup identified by Cattenoz et al. (2020). The violet dots represent the expression in Cattenoz et al. (2020) subgroups, the red dots, expression in the hemocytes from stage 6 embryos (E6 embryos), the green dots in the hemocytes from larval eye discs, the purple and orange dots in the hemocytes from larval brains at the indicated stages (f 3rd instar: feeding 3rd instar larvae; w 3rd instar: wandering 3rd instar larvae). The arrowheads (red, green, purple) indicate the strongest markers linking the tissue-specific hemocytes with the subgroups from Cattenoz et al. (2020).

For example, PM9 in Tattikota et al. dataset shares a significant number of markers with PL-0, PL-3, PL-vir1, PL-prolif, PL-Inos, and PL-robo2 in Cattenoz et al. dataset (Figure 2A). The subgroup presenting the highest number of markers with the highest enrichments is PL-prolif (Supplementary Figure S1A). The second closest subgroup is PL-robo2, which also displays markers with relatively high enrichments. However, PL-robo2/PM9 markers are expressed in most hemocyte subgroups while PL-prolif/PM9 markers are highly specific (Supplementary Figure S1B). At last, lineage predictions carried out in both studies suggest that PM9 and PL-prolif are sitting at the beginning of the developmental trajectories of the hemocytes (Cattenoz et al., 2020; Tattikota et al., 2020). Altogether, these data suggest that PM9 and PL-prolif represent the same subgroup.

The marker comparison shows a large correlation across studies for crystal cells and lamellocytes: 53 crystal cell markers and 265 lamellocyte markers are commonly found by Tattikota et al. and Cattenoz et al. (Figures 2D,E). All the markers previously used to label the crystal cells and the lamellocytes are present in the two datasets. Such tight correlation is expected for subgroups that present strong physiological differences compared to the plasmatocytes. Fu et al. disclosed a limited list of markers for crystal cells and lamellocytes; all of them are nevertheless present in the crystal cell and lamellocyte subgroups identified in two other datasets, further confirming the identity of these subgroups. The three studies consistently found a large subgroup of “unspecified plasmatocytes” presenting no distinctive marker and encompassing more than 50% of plasmatocytes (PM1 in Tattikota et al.; PL-0, 1, 2, and 3 in Cattenoz et al.; reservoir PM in Fu et al.), as well as several smaller subgroups enriched for specific markers: the proliferative, the antimicrobial, the posterior signaling center-like, the phagocytic and the secretory plasmatocytes (Figure 2B).

### Proliferative Plasmatocytes

The markers involved in mitosis, including Cyclin B (CycB) (Gavet and Pines, 2010), the phosphatase String (Stg) (Edgar and O’Farrell, 1990), the importin Pendulin (Pen) (Kussel and Frasch, 1995) and the kinase Polo (Sunkel and Glover, 1988) are enriched in clusters PM2 as well as PM9 in Tattikota et al., and in the PL-prolif cluster in Cattenoz et al. (Supplementary Figures S2A,B). These proliferative subgroups are positioned at the beginning of the developmental trajectories and give rise to the pool of unspecified plasmatocytes. The PL-prolif subgroup represents a hemocyte subset of the PL-Inos subgroup, which lacks the proliferative genes but expresses all the other markers of PL-prolif (Cattenoz et al., 2020), suggesting that PL-prolif and PL-Inos represent two states (dividing/quiescent) of the same subgroup.

### Antimicrobial Plasmatocytes

Two datasets contain subgroups of plasmatocytes enriched in transcripts involved in immune pathways: PL-AMP, PL-Rel and PL-vir1 in Cattenoz et al.; PM3, PM5, PM6, and PM7 in Tattikota et al. The comparison of the markers indicates a correlation between PL-Rel/PL-AMP and PM3/PM6/PM7 as well as between PL-vir1 and PM3/PM5 (Supplementary Figures S2C,D).

PM6/7 and PL-AMP are highly enriched for AMP expression [i.e., Cecropins A1, A2, and C (CecA1, CecA2, CecC), Diptericin B (DptB), Drosomycin (Drs), Metchnikowin (Mtk), Mtk-like (Mtkl)]. Of note, the AMPs Mtk, DptB, and Drs are enriched at higher levels in the dataset from Tattikota et al. than in the one from Cattenoz et al. This is likely due to the induction of these AMPs by clean wounding, a condition specific to the dataset from Tattikota et al. All these subgroups also express high levels of Matrix metalloproteinase 1 (Mmp1), involved in wound healing (Stevens and Page-McCaw, 2012). PM3 and PL-Rel express elements of the IMD and JNK pathways and lower levels of AMP, which suggests weaker or different mode of activation of the immune response. This may reflect different microenvironments and/or different intrinsic properties compared to PM6/7 and PL-AMP.

PM5 and PL-vir1 present common expression profiles, distinctive from the other subgroups. They are not enriched in AMPs (Supplementary Figure S2D) and seem specialized in xenobiotic detoxification, as suggested by the expression of the Ferritin 1 heavy chain homolog, the Ferritin 2 light chain homolog and the Multidrug resistance protein (Chahine and O’Donnell, 2009; Tang and Zhou, 2013).

### Posterior Signaling Center-Like Plasmatocytes

The comparison of the markers consistently identifies a subgroup of cells representing less than 1% of the hemocyte population across the three studies: PL-ImpL2 (Cattenoz et al., 2020), PM11 (Tattikota et al., 2020), and the primocytes (Fu et al., 2020) (Supplementary Figures S2E,F). This subgroup expresses typical markers of the posterior signaling center (PSC) present in the lymph gland, such as the transcription factors Knot (Kn) (Makki et al., 2010) and Antennapedia (Antp) (Mandal et al., 2007). The comparison of the markers of this subgroup with the PSC markers identified by the scRNAseq assay on the lymph gland (Cho et al., 2020) also identifies this subgroup as PSC-like plasmatocytes (Supplementary Figure S2G).

### Phagocytic Plasmatocytes and Secretory Plasmatocytes

The datasets from Fu et al. and from Cattenoz et al. identified phagocytic plasmatocytes, plasmatocytes secreting storage proteins and plasmatocytes secreting opsonins.

Phagocytic plasmatocytes: CAH7 + PM (Fu et al.) and PL-robo2 (Cattenoz *e*t al*.)* are enriched for the phagocytic receptor NimC2 (Kurucz et al., 2007), the cytoskeleton proteins Myoblast city (Erickson et al., 1997), the Tenascin accessory (Mosca et al., 2012), the transmembrane receptor Lipophorin receptor 2 (LpR2) and Mmp2 (Supplementary Figure S2H). Lineage predictions call for PL-robo2/CAH7 + PM being directly issued from the unspecified plasmatocytes and since the strongest markers for PL-robo2 are also enriched to a lower extent in a subset of unspecified plasmatocytes (PL-0/PL-2), PL-robo2 may specifically represent the phagocytic, active state, of this subset.

Secretory plasmatocytes: Lsp + PM (Fu et al.) and PL-Lsp (Cattenoz et al.) display a highly distinctive expression pattern of proteins secreted in the hemolymph such as Larval serum protein 1 alpha (Lsp1alpha), Larval serum protein 2 (Lsp2), Apolipophorin (Apolpp), and Odorant binding protein 99b (Obp99b) (Supplementary Figure S2I). All these proteins are mostly expressed by the fat body (Chintapalli et al., 2007) and serve as storage proteins (Telfer and Kunkel, 1991; Handke et al., 2013), suggesting common functions between the fat body and these secretory plasmatocytes.

At last, the thanacytes (Fu et al.) and PL-Pcd (Cattenoz et al.) express low levels of NimC1 and secrete the opsonins Thioester-containing protein 2 and 4 (Tep2 and Tep4) that promote the phagocytosis of bacteria and the activation of the Toll pathway (Dostalova et al., 2017; Supplementary Figure S2J). The thanacyte markers are consistently enriched in both PL-AMP and PL-Pcd, yet differ from PL-AMP by the lack of AMP production (Supplementary Figure S2J). Thus, PL-AMP may represent an activated state of PL-Pcd/thanacyte, in which the inflammatory pathways are triggered and antimicrobial peptides are secreted.

These three subgroups were not identified in Tattikota et al. dataset. Their markers are detected in some cells but these cells are not clustered together and are associated with the pool of unspecified plasmatocytes, with whom they share the majority of the markers.

### Lamellocytes

The datasets from Tattikota et al. and Cattenoz et al. include conditions promoting the differentiation of the lamellocytes and identify two subgroups. One of them expresses both plasmatocyte and lamellocyte markers and likely corresponds to plasmatocytes that are transdifferentiating into lamellocytes (LM1 in Tattikota et al., LM-2 in Cattenoz et al.). The second subgroup lacks most of the plasmatocyte markers and expresses the lamellocyte markers strongly (LM2 in Tattikota et al., LM-1 in Cattenoz et al.). For the sake of simplicity, we will call the first and the second subgroups intermediary and mature lamellocytes, respectively.

To identify the molecular pathways activated during lamellocyte differentiation, we carried out a regulon analysis using SCENIC (Aibar et al., 2017) on the dataset from wasp infested larvae generated by Cattenoz et al. (2020). This analysis relies on two steps. First, the genes presenting covariation are identified across the whole single cell dataset. Then, the promoters of the covariating genes are scanned for canonical transcription factor binding sites. The genes presenting similar expression profiles and carrying the same transcription factor binding site(s) are grouped in one regulon named after the transcription factor. This analysis highlights two regulons, Kayak (Kay) and Jun-related antigen (Jra), which regroup the targets of the two main transcription factors of the JNK pathway (reviewed in La Marca and Richardson, 2020; Figure 3A), known to promote the differentiation of lamellocytes (Tokusumi et al., 2009). We have pulled together the transcriptomes of all cells from the intermediary and mature lamellocytes from Cattenoz et al. dataset to generate pseudo-transcriptomes. The comparison of the pseudo-transcriptomes shows that the genes regulated by the JNK pathways are expressed at higher levels in the mature lamellocytes than in the intermediary ones (Figure 3B). Strikingly, this analysis also reveals for the first time seven novel regulons associated with lamellocyte differentiation: Cyclic-AMP response element binding protein B (CrebB), Forkhead box sub-group O (Foxo), REPTOR-binding partner (REPTOR-BP), Pannier (Pnr), Maf-S, Zinc-finger protein (Zif), and Checkpoint suppressor 1-like (CHES-1-like). These regulons are highly enriched in lamellocytes and display an enhanced activation from the intermediary lamellocytes to the mature lamellocytes (Figure 3A).

The regulons CrebB, Foxo and REPTOR-BP are involved in the maintenance of energy metabolism upon food restriction or molecular stress (Dionne et al., 2006; Iijima et al., 2009; Tiebe et al., 2015). They may be responsible for the induction of specific glucose transporters and the metabolic shift from lipolytic to glycolytic observed during the transdifferentiation of plasmatocytes into lamellocyte (Cattenoz et al., 2020; Tattikota et al., 2020). These pathways may also maintain the high metabolic activity of lamellocytes, while the metabolism of other organs is inhibited to privilege the immune response over the developmental processes (Bajgar et al., 2015; Dolezal, 2015). Maf-S along with Foxo regulate oxidative stress resistance (Rahman et al., 2013; Gumeni et al., 2019) and CHES-1-like is involved in the DNA-damage response (Busygina et al., 2006). Both oxidative stress and DNA damage can be associated with the high metabolism of lamellocytes, a phenomenon usually observed in cancerous cells in mammals (Moretton and Loizou, 2020).

### Hemocyte Populations in Other Single Cell Datasets

In mammals, macrophages are conditioned by their microenvironment, which leads to molecular signatures specific to the tissues in which they reside (van de Laar et al., 2016; Guilliams et al., 2020). To assess if specific hemocyte subgroups are associated to different tissues in *Drosophila*, we have screened tissue specific or embryonic scRNAseq datasets containing hemocytes (i.e., larval eye discs, larval brains, and stage 6 embryos) and compared their molecular signatures to the dataset of Cattenoz et al.

#### Hemocytes Associated With the Larval Eye Disc Resemble Unspecified Plasmatocytes

The scRNAseq analyses on larval eye discs (Ariss et al., 2018) reveal a small subgroup of cells in line with the hemocytes observed in close contact with this disc (Holz et al., 2003; Figure 4A and Supplementary Figures S3A,B). Since the eye disc associated hemocytes express a large panel of hemocyte markers, their pseudo-transcriptome was compared to the pseudo-transcriptome of each of the subgroups identified in Cattenoz et al. dataset using Pearson correlation coefficient. This analysis highlights a strong correlation between the eye disc associated hemocytes and unspecified plasmatocytes (PL-1/PL-3, Supplementary Figure S3C), which do not express strong markers. Moreover, the eye disc associated hemocytes do not express subgroup-exclusive markers, like Lsp1alpha in PL-Lsp or Kn and Tau in PL-ImpL2 (Figure 4D). Altogether, these evidence suggest that the eye disc associated hemocytes are unspecified plasmatocytes.

Next, to determine if the eye disc associated hemocytes represent a subgroup that was not defined in Cattenoz et al. dataset, we compared their pseudo-transcriptome to the combined pseudo-transcriptome of all subgroups from Cattenoz et al. (Supplementary Figure S4A). This comparison did not return a strong signature for the eye disc associated hemocytes (less than 10 genes specifically expressed in the eye disc hemocytes), which is concordant with the high correlations measured between almost all subgroups and the eye-associated hemocytes (Supplementary Figure S3C). Thus, at this level of resolution, the hemocytes in the eye disc cannot be distinguished from unspecified plasmatocytes.

One of the main characteristics of the unspecified plasmatocytes is the expression of Extra Cellular Matrix (ECM) components such as Pxn, which is necessary for the assembly of the basement membrane (Bhave et al., 2012). Pxn is indeed strongly expressed in the hemocytes associated to the eye disc and is detected at the basement membrane of the disc (Figure 4A). Similar analyses will reveal whether this feature is common to the hemocytes associated to the other imaginal tissues. Interestingly, larval hemocytes secrete ECM compounds to build the basement membrane of the ovaries and the fat body (Shahab et al., 2015; Van De Bor et al., 2015).

#### Hemocytes Associated With the Brain Resemble the Proliferative Plasmatocytes (PL-Inos, PL-Prolif)

Two scRNAseq datasets on the larval brain from 1st instar to 3rd instar larvae (Brunet Avalos et al., 2019; Cocanougher et al., 2019) report the presence of hemocytes (Supplementary Figure S3D), in line with the hemocytes observed in close contact with this tissue (Figure 4B). The comparison of the pseudo-transcriptomes of hemocytes from the 1st instar brain and of Cattenoz et al. subgroups highlights the highest correlation with the PL-prolif and PL-Inos subgroups (Supplementary Figure S3E). The correlation coefficients are much weaker than in the comparison with the eye disc associated hemocytes. In addition, the comparison of the pseudo-transcriptomes of brain associated hemocytes with the pseudo-transcriptome of all subgroups from Cattenoz et al. suggests a specific molecular signature for these hemocytes (Supplementary Figure S4B). PL-prolif markers are represented in the brain associated hemocytes, together with dozens of genes that are consistently expressed from 1st instar to 3rd instar larval brains (Figure 4D, Supplementary Table S1 and Supplementary Figure S4C). Altogether, these data suggest that the brain hemocytes represent a discrete subgroup with a specific molecular signature. The strong markers of PL-Inos/PL-prolif are preserved during development as they are observed from the 1st to the 3rd instar datasets (Figure 4D).

#### PSC-Like Plasmatocytes and Proliferative Plasmatocytes Resemble Prohemocytes From Stage 6 Embryos

The single cell analyses suggest that the larval hemocytes originate from the proliferative subgroup. We therefore predicted that the prohemocytes present at the early embryonic stages may resemble this subgroup and compared the larval dataset from Cattenoz et al. with the one obtained from scRNAseq on embryos at stage 6 (Supplementary Figure S3F; Karaiskos et al., 2017). At this stage, the cells of the procephalic mesoderm express the earliest hemocyte-specific transcription factors Glial Cell Missing/Glial Cell Deficient (Gcm) and Serpent (Srp) (Figure 4C; Sam et al., 1996; Bernardoni et al., 1997; Waltzer et al., 2003). The comparison of the pseudo-transcriptomes indeed shows that the prohemocytes from stage 6 embryos present the highest correlation with the subgroups PL-prolif/PL-Inos (i.e., proliferative plasmatocytes) and PL-ImpL2 (i.e., PSC-like plasmatocytes), and express the strongest markers of these subgroups (Figure 4D and Supplementary Figure S3G).

## Discussion

The comparative analysis of the scRNAseq datasets confirms the diversity of the immune cells present in the *Drosophila* larva. Molecular features consistently found across the studies allow the identification of robust plasmatocyte subgroups in addition to crystal cells and lamellocytes. The proliferative subgroup resembles the prohemocytes present in the early embryonic stages and the plasmatocytes associated with different larval tissues seem to present specific features.

### Identification of Robust Subgroups

The present analysis identifies common subgroups with specific potentials (proliferation, AMP production, PSC-like, phagocytosis, secretion, crystal cells and lamellocytes) or lacking specific properties (unspecified plasmatocytes) (summarized in Figures 2B,C).

The proliferative plasmatocytes seem the sole source of larval hemocytes, including the crystal cells (Cattenoz et al., 2020; Tattikota et al., 2020). This subgroup may represent cells retaining a progenitor state, in line with their resemblance with the molecular signature of the hemocyte progenitors present in the early embryo. Lineage predictions suggest that the proliferative subgroup generates the unspecified plasmatocytes, which constitute more than 50% of the hemocytes and represent intermediary states between proliferative and differentiated cells (Cattenoz et al., 2020; Tattikota et al., 2020). The unspecified plasmatocytes seem to constitute the majority of the hemocytes associated with the eye discs, where they produce ECM proteins. The antimicrobial subgroup is the largest specified subgroup. It encompasses plasmatocytes enriched in transcripts coding for proteins associated to the immune response, including elements of the IMD and the Toll pathways and AMPs. The PSC-like subgroup displays a signature highly similar to that of the PSC in the lymph gland. Future studies will determine whether this subgroup plays a similar signaling role in the hemolymph and/or in specific niches. The phagocytic plasmatocytes are enriched for markers involved in engulfment, phagocytosis and actin remodeling. The secretory plasmatocytes are subdivided into two subgroups, one secreting storage proteins and the other secreting opsonins. The phagocytic and the storage protein secreting subgroups resemble strongly the unspecified plasmatocytes, as the number of specific markers is quite limited. The lack of a strong distinctive signature suggests that these cells may be associated with specific microenvironments/challenges, which would induce the expression of proteins on-demand, without drastically affecting hemocyte properties and identity. In other terms, we hypothesize that these two clusters are of common nature and that their differences rely on different “nurture.”

In addition to the plasmatocyte subgroups, the three studies identified lamellocyte and crystal cell subgroups. Both subgroups are characterized by a specific list of markers, suggesting a well-defined nature of these cells. Some markers are already known (Rizki and Rizki, 1959; Braun et al., 1997; Irving et al., 2005; Rus et al., 2006; Honti et al., 2009; Binggeli et al., 2014), some are new. The datasets from Tattikota et al. and Cattenoz et al. include similar experimental immune challenges (i.e., wasp infestation) to activate the production of lamellocytes and display two subgroups of lamellocytes, one intermediary and one mature. The regulons analysis identifies the transcription factors involved in lamellocyte transdifferentiation/functions. These include the transcription factors of the JNK pathway known to promote lamellocyte differentiation and seven novel transcription factors potentially required for lamellocytes maturation. Further investigations will determine the roles of these transcription factors in the differentiation of the lamellocytes.

### Advantages and Biases of the Computational Approach

The comparison of the different hemocyte scRNAseq datasets allows the identification of common molecular signatures, despite the heterogeneity of the experimental procedures. This straightforward approach is based on the marker lists provided by each study and is not computationally demanding. The downside is that it highlights only the subgroups presenting the strongest markers and biologically relevant minor variations can be hidden by the differences between the parameters chosen for the computational analysis. This is the case of the secretory and phagocytic subgroups, which were not identified in Tattikota et al. (2020). In addition to the different analytic parameters, the absence of these subgroups may also be explained by the different experimental conditions. Tattikota et al. dataset includes three experimental conditions with a majority of the cells coming from wounded larvae. Such set up may favor the identification of subgroups specific to wounded conditions at the expense of subgroups present in steady state conditions.

The molecular signature of the subgroups defined in Cattenoz et al. have been used to determine the identity of hemocytes present in the scRNAseq from larval eye discs and brains as well as from stage 6 embryos. The strong molecular signature of the hemocytes permits a clear distinction of these cells within each dataset and then the comparison with the hemocyte subgroups’ signatures allows the identification of the closest subgroups. Typically, the hemocytes associated with the brain display the distinctive profile of the small, proliferative, subgroup. The main caveat of this analysis is related to the limited number of hemocytes recovered in assay (<100 cells in each dataset). We cannot exclude that discrete subgroups were not found because of their low frequencies in the specific tissue. This may notably be the case of the hemocytes associated with the eye discs, which display markers of the large subgroup of unspecified plasmatocytes. In addition, the dissection procedure may remove the less tightly associated cells, rendering our interpretation incomplete. The development of *ad hoc* protocols to analyze the hemocytes in the whole animal will help significantly.

The comparison of scRNAseq datasets brings robustness to the precise information on the cellular diversity gained with a single dataset. However, this approach is highly dependent on the analysis pipelines followed within each study. Since the number of studies using scRNAseq are currently booming and in parallel, new analytical tools are constantly generated, the comparability between datasets is not always straightforward (Vieth et al., 2019; Gao et al., 2020). Generating a universal pipeline of scRNAseq analysis will be highly valuable as it will facilitate the comparison between datasets.

In sum, the comparison of the three scRNAseq datasets allows the definition of common features and robust subgroups, an important asset to understand the biology of hemocyte heterogeneity. Some subgroups show a distinctive transcriptomic signature, while others present many shared markers, suggesting an origin according to nature and nurture, respectively. Increasing evidence highlight the influence of the microenvironment in governing the gene expression profile in mammalian tissue-specific macrophages, despite their common embryonic origin (Gautier et al., 2012; Lavin et al., 2014). We expect that similar mechanisms in *Drosophila* lead to the differentiation of specific subgroups. Future lineage tracing assays will help assessing the relative impact of intrinsic and environmental cues on the presence of the different subgroups as well as their stability. The generation of subgroup specific drivers will make it possible to assess the role of the different subgroups in physiological and pathological conditions. Finally, bulk RNAseq of distinct subgroups (sorted according to the markers from the scRNAseq) will generate deeper molecular signatures. The combination of more refined experimental and analytical approaches will determine with higher precision the similarities and differences between subgroups and reveal the function of the subgroups. This will contribute to unravel the heterogeneity and the biology of the macrophage populations.

## Data Availability Statement

The data was deposited in the ArrayExpress database at EMBLEBI (www.ebi.ac.uk/arrayexpress) under the accession number E-MTAB-8698.

## Author Contributions

PC and AG: conceptualization, investigation, writing—original draft, funding acquisition, and supervision. PC, AG, AP, and SM: methodology and writing—review and editing. AG: resources. All authors contributed to the article and approved the submitted version.

## Conflict of Interest

The authors declare that the research was conducted in the absence of any commercial or financial relationships that could be construed as a potential conflict of interest.

## Footnotes

1 www.ebi.ac.uk/arrayexpress

2 https://satijalab.org/seurat/v3.2/pbmc3k_tutorial.html

3 https://pyscenic.readthedocs.io/en/latest/

4 www.ebi.ac.uk/arrayexpress

5 cistarget/motif2tf/motifs-v8-nr.flybase-m0.001-o0.0.tbl

6 cistarget/databases/drosophila_melanogaster/dm6/flybase_r6.02/mc8nr/gene_based/dm6-5kb-upstream-full-tx-11species.mc8nr.feather
