# Supplementary material for: Toward a Consensus in the Repertoire of Hemocytes Identified in Drosophila
Source: Front Cell Dev Biol. 2021 Mar 4;9:643712. doi: 10.3389/fcell.2021.643712 (PMC7969988; doi:10.3389/fcell.2021.643712)
Supplement: Supplementary file 1 [file Data_Sheet_1.pdf]

Supplementary files:

Supplementary Figure S1

Supplementary Figure S2

Supplementary Figure S3

Supplementary Figure S4

Supplementary Table S1

Bibliography supplementary file

Supplementary Figures

Supplementary Figure S1

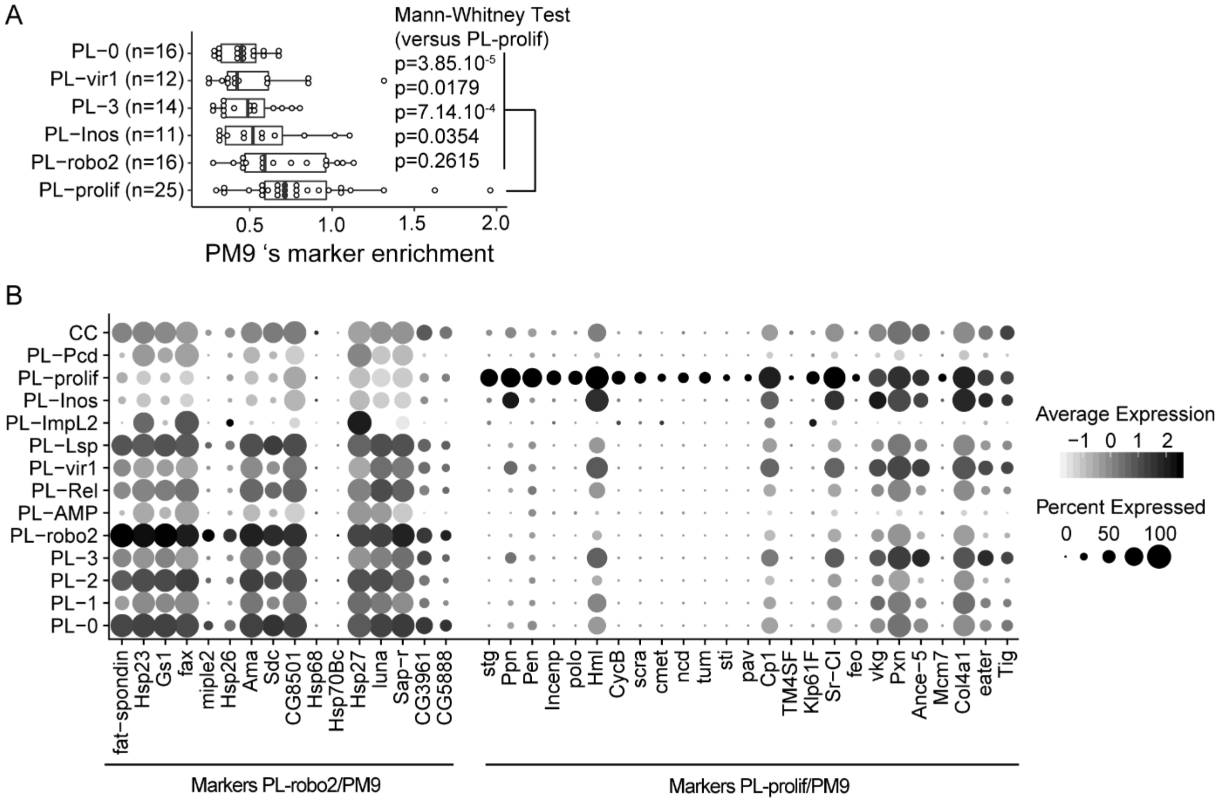

Supplementary Figure S1: Determining equivalence between PM9 in Tattikota *et al.* dataset and PL-0, PL-vir1, PL-3, PL-Inos, PL-robo2 and PL-prolif in Cattenoz *et al.* dataset.

A) *Boxplot representing the enrichment levels of the common markers between PM9 (Tattikota et al., 2020) and PL-0, PL-vir1, PL-3, PL-Inos, PL-robo2 and PL-prolif (Cattenoz et al., 2020). The number of markers common to each of the subgroups from Cattenoz et al. and the PM9 subgroup is indicated between parentheses. Note that PL-prolif presents the highest number of markers with the highest enrichment. This subgroup is significantly different from all the other subgroups except PL-robo2. The p-values were estimated using the two-tails Mann-Whitney non-parametric test for independent samples. The boxes encompass the 2nd and 3rd quartiles and the median is indicated with a thick grey line.*

B) *Dot plot representing the percentage of cells and the expression levels of the common markers between PM9 and PL-robo2 or PL-prolif. The expression levels are represented with a gradient of grey intensity and the percentage of cells is represented by the size of the dot. Note that most PM9/PL-robo2 markers are also expressed in most subgroups (shown on the y axis) while the PM9/PL-prolif markers display high specificity for PL-prolif subgroup.*

Supplementary Figure S2

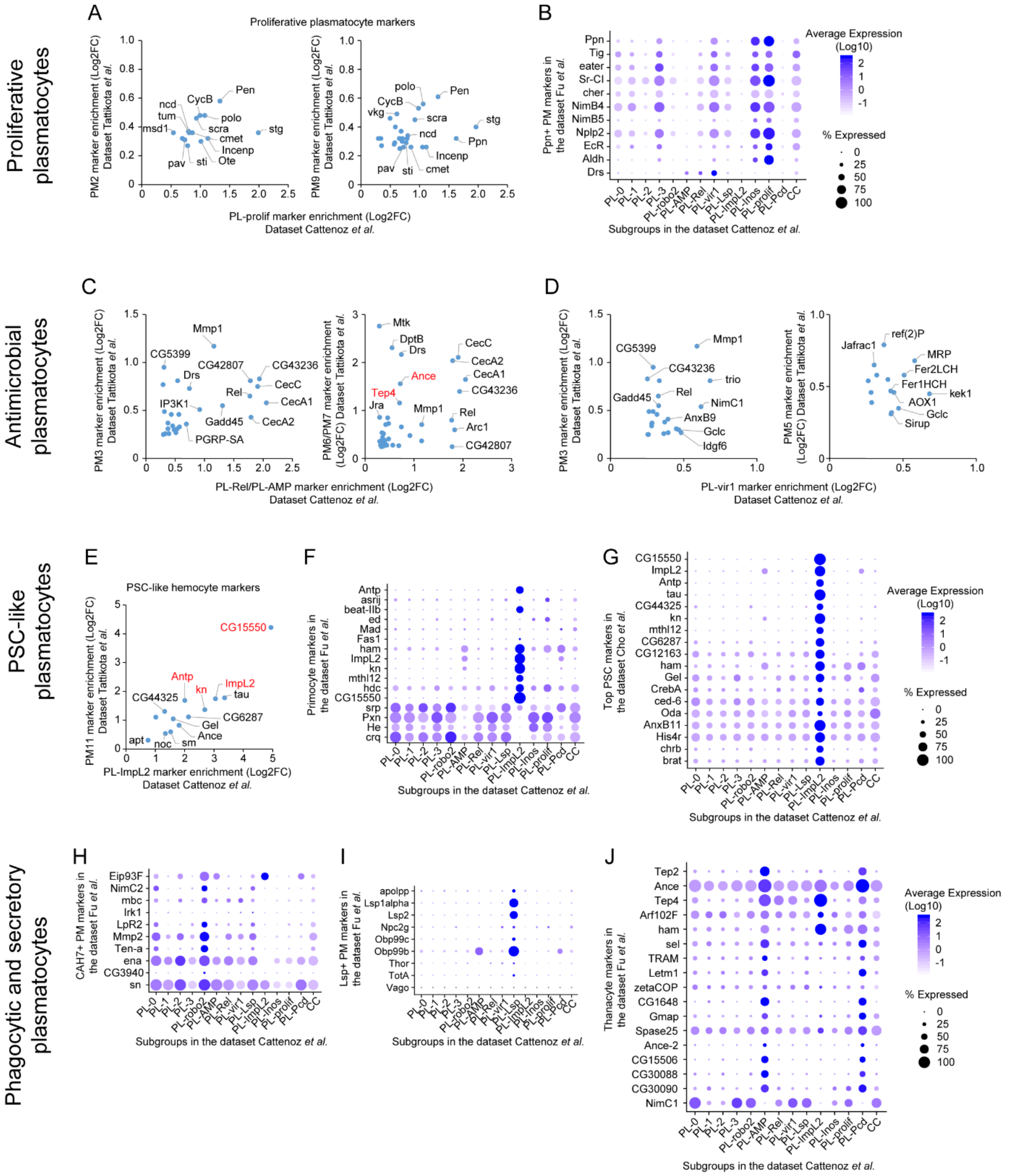

Supplementary Figure S2: Identification of the proliferative, antimicrobial, PSC-like, phagocytic and secretory subgroups.

A) *Scatter plots comparing the enrichment levels of the markers common between PM2 (left panel) or PM9 (right panel) (Tattikota et al., 2020) and PL-prolif (Cattenoz et al., 2020). Note that the markers involved in mitosis present the highest levels of enrichment (i.e. Pen, polo, stg, CycB).*

B) *Dot plot representing the percentage of cells and the expression levels of the markers in the Ppn+ PM subgroup (Fu et al., 2020) and in the subgroups identified in (Cattenoz et al., 2020). The expression*

levels are represented with a gradient of violet levels and the percentage of cells is represented by the size of the dot. Note that the markers are mostly expressed in PL-Inos and PL-prolif.

C) *Scatter plots comparing the enrichment of the markers common between PM3 (left panel) or PM6/PM7 (right panel) (Tattikota et al., 2020) and PL-Rel/PL-AMP (Cattenoz et al., 2020).*

D) *Scatter plots comparing the enrichment of the markers common between PM3 (left panel) or PM5 (right panel) (Tattikota et al., 2020) and PL-vir1 (Cattenoz et al., 2020).*

E) *Scatter plot comparing the enrichment of the markers common between PM11 (Tattikota et al., 2020) and PL-ImpL2 (Cattenoz et al., 2020). Genes annotated in red are also described in the primocyte subgroup in the dataset from Fu et al..*

F) *Dot plot representing the percentage of cells and the expression levels of the primocytes markers (Fu et al., 2020) in the subgroups identified by Cattenoz et al. (Cattenoz et al., 2020). The markers crq, He, Pxn and srp are inhibited in primocytes (Fu et al., 2020) and in PL-ImpL2 (Cattenoz et al., 2020).*

G) *Dot plot representing the percentage of cells and the expression levels of the markers of the lymph gland PSC (Cho et al., 2020) in the subgroups identified by Cattenoz et al. (Cattenoz et al., 2020). The subgroup PL-ImpL2 is enriched for all the markers.*

H-J) *Dot plots representing the percentage of cells and the expression levels of the markers of the CAH7+ PM subgroup (**H**), of the Lsp+ PM subgroup (**I**) or of the thanacytes (**J**) from (Fu et al., 2020) in the subgroups identified by (Cattenoz et al., 2020). Note that these subgroups were not found in the dataset from Tattikota et al..*

Supplementary Figure S3

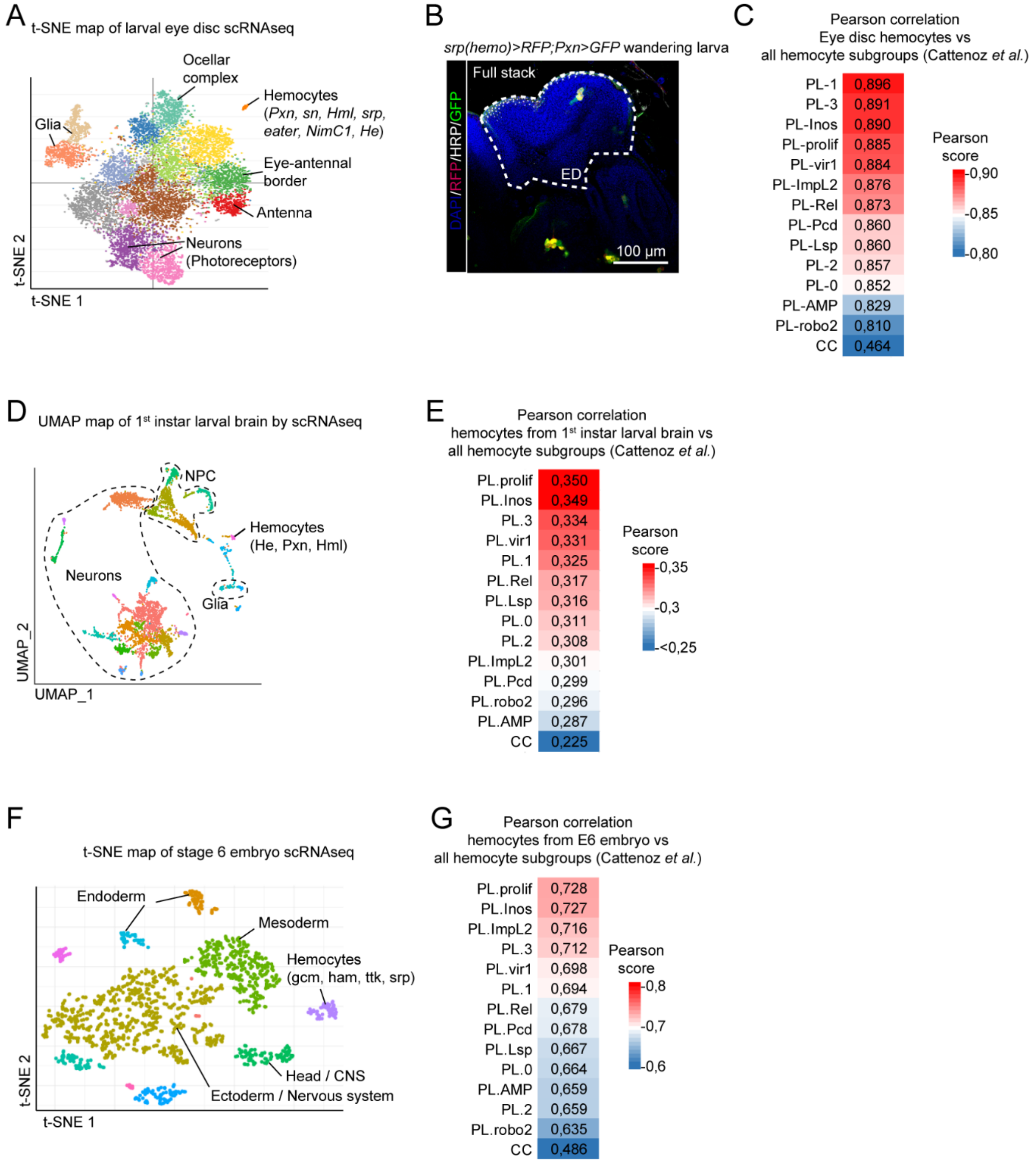

Supplementary Figure S3: Molecular signatures of the hemocytes from larval eye discs, larval brains and stage 6 embryos.

A) *tSNE map of the scRNAseq dataset from 3rd larval instar eye discs (modified from https://portals.broadinstitute.org/single_cell (Ariss et al., 2018)). The main markers of the hemocyte subgroups are annotated on the graph.*

B) *Eye disc (ED) from srp(hemo)>RFP;Pxn>GFP larva. The immunolabelling assay used anti-RFP (in red) and anti-GFP (in green) to label hemocytes and anti-HRP to label neurons (in grey) (Jan and Jan, 1982). The nuclei were labelled with DAPI (blue). The scale bar represent 100 µm.*

C) *Pearson correlation coefficient between the pseudo-transcriptome from the larval eye discs associated hemocytes and each hemocyte subgroup from Cattenoz et al. dataset. The coefficients are colour coded with a gradient from blue (R <= 0,80) to red (R = 0,90).*

D) *UMAP map of the scRNAseq dataset on brains from 1st instar larvae (Brunet Avalos et al., 2019). The main markers of the hemocyte subgroups are annotated on the graph.*

E) *Pearson correlation coefficient (R) between the pseudo-transcriptome from the 1st instar larval brains associated hemocytes and each hemocyte subgroup from Cattenoz et al. dataset. The coefficients are colour coded with a gradient from blue (R <= 0,60) to red (R=0,80).*

F) *tSNE map of the scRNAseq dataset on stage 6 embryos (modified from https://shiny.mdc-berlin.de/DVEX/, (Karaiskos et al., 2017)). The main markers of the hemocyte subgroups are annotated on the graph.*

G) *Pearson correlation coefficient (R) between the pseudo-transcriptome from stage 6 embryo hemocytes and each hemocyte subgroup from Cattenoz et al. dataset. The coefficients are colour coded with a gradient from blue (R <= 0,60) to red (R = 0,80).*

Supplementary Figure S4

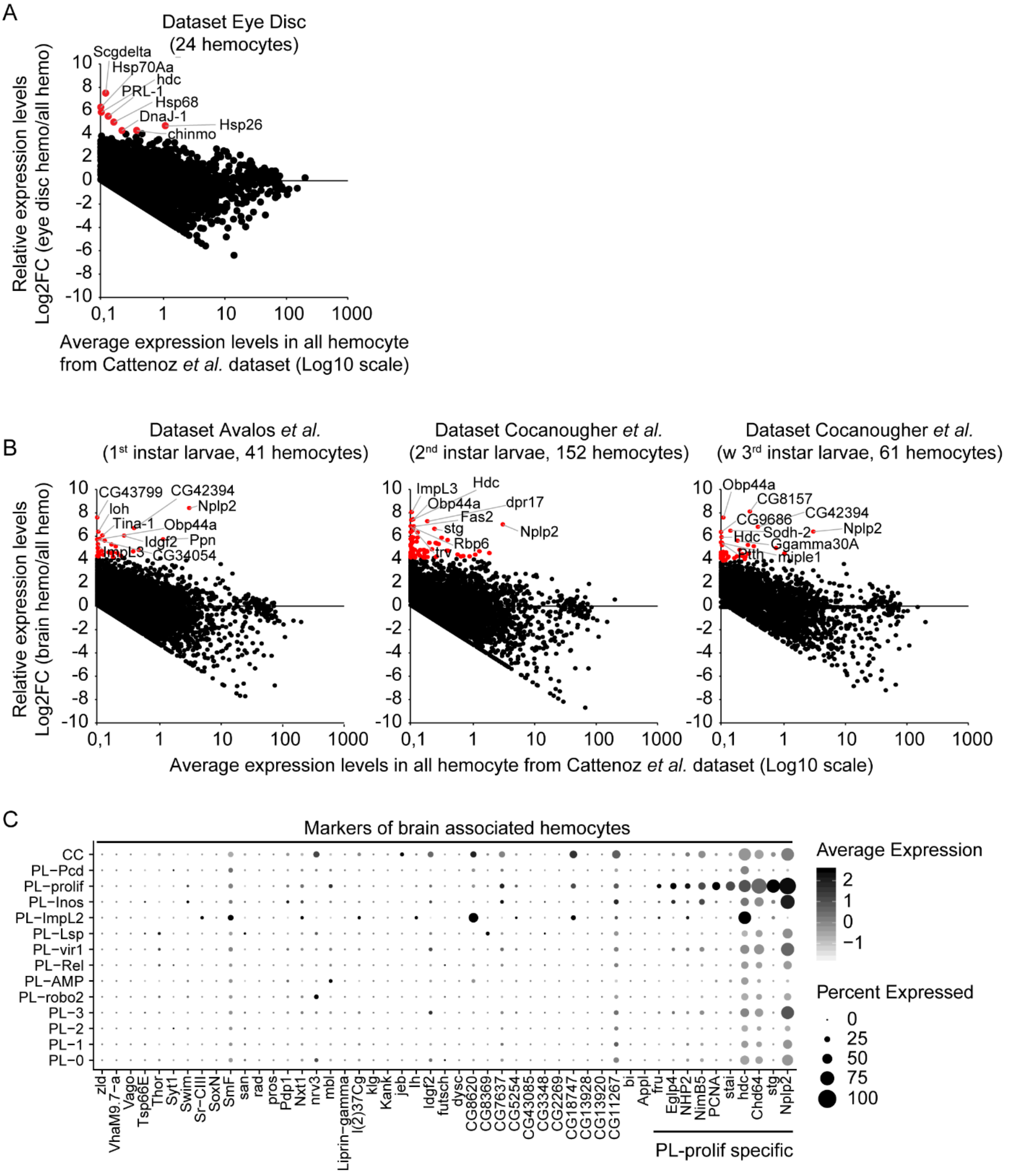

Supplementary Figure S4: Molecular signatures of the hemocytes from larval eye discs, larval brains and stage 6 embryos.

A,B*) Scatter plots comparing the hemocyte pseudo-transcriptome from Cattenoz et al. dataset with* ***(A)*** *the pseudo-transcriptomes of eye disc associated hemocytes (Ariss et al., 2018) and with* ***(B)*** *the brain associated hemocytes from Avalos et al. (brain from 1st instar larvae (Brunet Avalos et al., 2019), left panel) and Cocanougher et al. datasets (from 2nd instar and wandering 3rd instar larvae (Cocanougher et al., 2019), middle and right panels, respectively). The x-axis represents the average expression levels in all hemocytes (in Log10 scale), the y-axis represents the Log2 fold change (Log2FC) of the ratio [(levels in brain hemocytes +0.1)/ (levels in all hemocytes + 0.1)]. Genes enriched in eye disc or brain hemocytes*

present positive Log2FC. The top ones (Log2FC > 4) are highlighted in red (8 markers for Ariss et al., 31 markers for Avalos et al., 81 markers for 2nd instar larvae, 47 markers for w 3rd instar larvae in Cocanougher et al.) and the top 10 are annotated on the graphs. The markers specific to brain associated hemocytes are listed in ***Supplementary Table S1****.*

C) *Dot plot representing the percentage of cells and the levels of the common markers between the hemocytes associated with the larval brain and the hemocyte subgroups determined by Cattenoz et al.. The 49 markers were selected for enrichment across the three brain scRNAseq datasets (Log2FC >4 in a least one dataset and >2 in the two other ones). The main markers of PL-prolif are enriched in the brain associated hemocytes (dots on the right, annotated as PL-prolif specific) suggesting that the brain associated hemocytes are PL-prolif hemocytes. Moreover, a significant number of markers seem specific to the brain associated hemocytes (lowly expressed across all subgroups), suggesting that the brain associated hemocytes represent a specific subset of proliferative hemocytes poorly represented in Cattenoz et al. dataset.*

Supplementary Table S1

Symbol	Fbgn	Pseudotranscriptome steady state hemocyte (Cattenoz *et al.*)	L2FC L1 brain (Avalos)	L2FC L2 brain (Coucanougher)	L2FC L3 brain (Coucanougher)
**Ank2**	FBgn0261788	0,0000	2,08	4,99	2,60
**Appl**	FBgn0000108	0,0000	2,99	4,38	4,41
**Argk**	FBgn0000116	0,0081	4,25	4,42	4,70
**bru3**	FBgn0264001	0,0044	4,11	4,48	3,61
**CG13928**	FBgn0035246	0,0000	2,92	3,36	4,64
**CG14253**	FBgn0039467	0,0256	4,24	3,64	2,57
**CG15093**	FBgn0034390	0,0805	4,45	3,68	4,31
**CG15211**	FBgn0030234	0,0327	5,45	3,08	4,28
**CG17124**	FBgn0032297	0,0028	2,28	4,84	2,70
**CG2269**	FBgn0033484	0,0000	3,28	4,42	2,57
**CG42394**	FBgn0259740	0,2954	6,52	5,51	6,81
**CG4250**	FBgn0034761	0,9617	3,94	3,86	4,56
**CG43085**	FBgn0262531	0,0000	2,91	3,07	4,40
**CG43324**	FBgn0263029	0,0169	2,83	2,46	4,52
**CG6329**	FBgn0033872	0,0021	3,17	5,53	3,49
**CG7646**	FBgn0036926	0,0649	2,93	2,60	4,16
**CG8157**	FBgn0034010	0,1930	10,26	10,09	8,12
**CG8369**	FBgn0040532	0,0166	2,62	4,19	3,08
**CG9686**	FBgn0030158	0,0000	3,25	3,59	6,38
**chinmo**	FBgn0086758	0,2822	4,53	4,61	3,84
**Cngl**	FBgn0263257	0,0000	3,33	4,18	4,14
**cpo**	FBgn0263995	0,0079	3,82	6,34	4,10
**cpx**	FBgn0041605	0,0163	3,49	4,69	3,11
**Dbi**	FBgn0010387	0,0041	2,68	3,85	5,26
**dpr17**	FBgn0051361	0,0855	2,44	7,11	3,46
**Drat**	FBgn0033188	0,2168	2,00	5,70	2,95
**dysc**	FBgn0264006	0,0000	2,47	4,17	3,79
**Fas2**	FBgn0000635	0,0120	2,76	6,68	2,69
**fne**	FBgn0086675	0,0287	3,35	5,72	4,19
**fru**	FBgn0004652	0,0287	3,19	6,15	2,37
**futsch**	FBgn0259108	0,0040	2,27	5,77	4,40
**Gat**	FBgn0039915	0,0083	4,47	2,86	2,58
**Ggamma30A**	FBgn0267252	0,0858	3,95	4,07	5,64
**Gs2**	FBgn0001145	0,1641	3,26	4,13	3,37
**GstD3**	FBgn0010039	0,0182	4,58	3,26	3,98
**Hdc**	FBgn0010113	0,0021	4,82	7,21	5,95
**Idgf2**	FBgn0020415	0,1731	5,87	2,69	4,55
**inaE**	FBgn0261244	0,0167	2,44	4,03	2,36
**Jafrac1**	FBgn0040309	0,1000	4,95	5,28	4,86
**jeb**	FBgn0086677	0,0029	2,62	4,81	3,60
**Liprin-gamma**	FBgn0034720	0,0000	2,11	6,18	4,12
**mbl**	FBgn0265487	0,1207	2,72	4,73	4,51
**MFS9**	FBgn0038799	0,0000	3,89	4,09	3,49
**miple1**	FBgn0027111	0,0035	4,47	3,20	5,46
**Nplp2**	FBgn0287423	3,0068	8,22	6,83	6,41
**nrv3**	FBgn0032946	0,1826	2,89	5,17	4,53
**Nxt1**	FBgn0028411	0,1479	3,14	2,23	4,27
**Obp44a**	FBgn0033268	0,0093	5,60	7,28	7,61
**Ptp99A**	FBgn0004369	0,0834	2,75	4,52	2,26
**Rbp6**	FBgn0260943	0,0082	3,32	6,59	3,93
**Sap47**	FBgn0013334	0,4757	2,24	4,29	2,79
**Sodh-2**	FBgn0022359	0,0426	3,88	4,67	6,49
**stg**	FBgn0003525	0,1431	4,16	6,46	2,46
**Tina-1**	FBgn0035083	0,0215	5,90	3,15	4,51
**zld**	FBgn0259789	0,0000	2,37	4,21	3,56

Supplementary Table S1: List of markers enriched in hemocytes associated with the larval brain

The 1st column indicates the symbol, the 2nd one the Flybase unique identifier (Fbgn), the 3rd the average expression levels in the hemocytes from Cattenoz et al.. 4th to 6th columns indicate the log2 fold change (L2FC) of the ratio [(levels in brain hemocytes +0.1)/ (levels in all hemocytes + 0.1)] for hemocytes associated with L1, L2 and L3 brains, respectively. The markers were selected for enrichment across the three brain scRNAseq datasets (Log2FC >4 in a least one dataset and >2 in the two other ones).

Bibliography Supplementary file

Ariss, M.M., Islam, A., Critcher, M., Zappia, M.P., and Frolov, M.V. (2018). Single cell RNA-sequencing identifies a metabolic aspect of apoptosis in Rbf mutant. *Nat Commun* 9(1)**,** 5024. doi: 10.1038/s41467-018-07540-z.

Brunet Avalos, C., Maier, G.L., Bruggmann, R., and Sprecher, S.G. (2019). Single cell transcriptome atlas of the Drosophila larval brain. *Elife* 8. doi: 10.7554/eLife.50354.

Cattenoz, P.B., Sakr, R., Pavlidaki, A., Delaporte, C., Riba, A., Molina, N., et al. (2020). Temporal specificity and heterogeneity of Drosophila immune cells. *EMBO J* 39(12)**,** e104486. doi: 10.15252/embj.2020104486.

Cho, B., Yoon, S.H., Lee, D., Koranteng, F., Tattikota, S.G., Cha, N., et al. (2020). Single-cell transcriptome maps of myeloid blood cell lineages in Drosophila. *Nat Commun* 11(1)**,** 4483. doi: 10.1038/s41467-020-18135-y.

Cocanougher, B., Wittenbach, J., Long, X.S., Kohn, A., Norekian, T., Yan, J., et al. (2019). "Comparative single-cell transcriptomics of complete insect nervous systems". bioRxiv).

Fu, Y., Huang, X., Zhang, P., van de Leemput, J., and Han, Z. (2020). Single-cell RNA sequencing identifies novel cell types in Drosophila blood. *J Genet Genomics* 47(4)**,** 175-186. doi: 10.1016/j.jgg.2020.02.004.

Jan, L.Y., and Jan, Y.N. (1982). Antibodies to Horseradish-Peroxidase as Specific Neuronal Markers in Drosophila and in Grasshopper Embryos. *Proceedings of the National Academy of Sciences of the United States of America-Biological Sciences* 79(8)**,** 2700-2704. doi: DOI 10.1073/pnas.79.8.2700.

Tattikota, S.G., Cho, B., Liu, Y., Hu, Y., Barrera, V., Steinbaugh, M.J., et al. (2020). A single-cell survey of Drosophila blood. *Elife* 9. doi: 10.7554/eLife.54818.
